# Pregnant women’s awareness, perception, and acceptability of COVID-19 vaccine attending antenatal clinics in Bharatpur, Nepal

**DOI:** 10.1371/journal.pone.0278694

**Published:** 2023-03-15

**Authors:** Radha Dhakal, Sushma Shapkota, Parita Shrestha, Prativa Adhikari, Shobhana Nepal

**Affiliations:** 1 Department of Nursing, Shree Medical and Technical College, Bharatpur Chitwan, Nepal; 2 Department of Public Health, Shree Medical and Technical College, Bharatpur Chitwan, Nepal; 3 Department of Nursing, Chitwan Medical College, Bharatpur Chitwan, Nepal; The 8th Medical Center of PLA General Hospital, CHINA

## Abstract

**Background:**

Vaccine is the cost-effective and reliable public health intervention to combat the emerging COVID-19 pandemic. The vaccination is considered safe and effective at any stage of pregnancy; however, pregnant women show more vaccine hesitation than the general population. This study aims to assess pregnant women’s awareness, perception, and acceptability of COVID-19 vaccine attending antenatal clinics.

**Methods:**

An institutional-based cross-sectional analytical study design was used to assess the acceptance of the COVID-19 vaccine and associated factors among pregnant women between Feb-1 to March-30–2022 at antenatal clinics of Bharatpur Chitwan using systematic random sampling. A semi-structured interview schedule was used to collect data from 644 respondents. Collected data were analysed using descriptive and inferential statistics like the Pearson chi-square test and logistic regression analysis.

**Results:**

The COVID-19 vaccine acceptance was found to be 22% and ethnicity (AOR = 1.826; 95% CI = 1.215–2.745), education level (AOR = 1.773; 95%CI = 1.025–3,068;), history of COVID-19 infection (AOR = 3.63; 95% CI = 1.323–9.956;), number of child (AOR = 5.021; 95% CI 1.989–12.677;), trimester (week of pregnancy) (AOR = 2.437; 95% CI 1.107–5.366) and level of perception (AOR = 2.152; 95% CI 1.109–4.178) were found to be statistically significant for acceptance of COVID-19 vaccine among pregnant mother.

**Conclusions:**

In this study, low levels of vaccine acceptance were found. Several influential factors like occupation, history of COVID-19 infection, number of pregnancies, week of gestation, and level of attitude were found to be significant for acceptance of COVID-19 vaccine among pregnant women. Everyone needs vaccine acceptance to get herd immunity and reduce the COVID-19 infection. But Vaccine hesitancy is one of the significant threats to the COVID-19 rollout and successful pandemic mitigation. Therefore, properly disseminating information and removing misperceptions about the COVID-19 vaccine is necessary to raise the acceptance.

## Introduction

The COVID-19 pandemic constitutes the most extensive global public health crisis in a century, with formidable health and socioeconomic challenges that have imposed enormous morbidity and mortality burdens on the general population [1,2]. The ideal approach to address emerging infection in an epidemic and pandemic is prevention through social mechanisms and vaccination [3]. Vaccines are one of the most reliable and cost-effective public health interventions ever implemented, saving millions of lives each year [4,5]. Vaccinated people are protected from getting the disease and breaking any chains of transmission [6]. Broad distribution and acceptance of vaccines are required to achieve herd immunity and expedite the end of the pandemic [7]. One of the significant threats to the COVID-19 vaccine rollout and successful pandemic mitigation is vaccine hesitancy [8]. Vaccine hesitancy as it is difficult to accept or outright refuse vaccines, despite their availability [9]. Several general factors also influence the reluctance to vaccination, including experience with vaccines, knowledge, risk perception, perceived importance of vaccination, religious and moral conviction [10].

Globally an estimated 211 million pregnancies occur every year [11]. In the context of COVID-19 infection, pregnancy is thought to be associated with a higher burden of maternal mortality and morbidity due to the physiologic cardiovascular, respiratory, and immunological adaptations of gestation. Pregnant women have a higher risk of becoming unwell with COVID-19 in the third trimester of pregnancy [12,13]. COVID-19 infections might also be associated with an increased risk of pregnancy complications such as preterm birth and caesarian section [14]. Pregnant women usually have a lower willingness and more concerns about vaccination than the general population [15]. Despite remarkable advances in vaccine research and development, vaccine hesitancy has been recognized as a public health threat [16].

The vaccine is considered safe and effective at any stage of pregnancy, but vaccine hesitancy has been found more in pregnant women than in the general population. Therefore, it is critical to recognize vaccine-hesitant factors, understand the causes of their hesitancies, and develop suitable strategies to address this. Everyone needs vaccine acceptance to get herd immunity and reduce the COVID-19 infection. So, researcher aims to identify the factors like awareness, perception, and acceptability of COVID-19 vaccine among the pregnant women attending antenatal clinics.

## Materials and methods

### Research design and research setting

An institutional-based analytical cross-sectional research design was used to assess pregnant women’s awareness, perception, and acceptability of COVID-19 vaccine attending antenatal clinics between Feb-1 to March—30–2022 at ANC clinics of Bharatpur Chitwan. Bharatpur is the district headquarter of the Chitwan District and a separate Metropolitan authority, with a population of 199,867and the proximity to Kathmandu (146 km) [17]. Safe motherhood programs are run by District Hospital Bharatpur, Chitwan Medical College (CMC) Pvt. Ltd, College of Medical Sciences (CMS), and Manakamana Hospital Pvt. Ltd. These hospitals are provided antenatal care and delivery service for pregnant mothers free of charge. In one month of data record review, the total number of pregnant women who came for ANC check-ups within four hospitals was 2790. In addition,1300 ANC attendees visited in district hospital Bharatpur, 650 in CMC, 480 in CMS, and 360 in Manakamana hospital. The researcher randomly selected two hospitals, Bharatpur and CMC, as a study setting.

#### Population

Pregnant women who received antenatal care from selected hospitals were chosen as the study population. Inclusion criteria were pregnant women aged 18 years or above; voluntary agreement to participate in the present study. In addition, pregnant women who were critically ill, have a documented history of mental illness and hearing impairment, and cannot provide the required information were excluded.

#### Sample size

The sample size was calculated based on 42% [18].

Sample size (n) = Zα_/2_^2^ *p*q / e^2^,

Here, zα = 1.96

Error (e) = 4% = 0.04%

Prevalence (p) = 42% = 0.42

q = (1—p) = 1–0.42 = 0.58

By using formula, the sample size is calculated as

Sample size (n) = (1.96) ^2^*0.42*0.58/ (0.04) ^2^

= 0.935581376/0.0016

= 584.7

Adding 10% non-response rate = 585+59 = 644

Required sample size was 644.

#### Sampling technique

At first, out of four safe motherhood programs implemented hospitals (Bharatpur district hospital, CMC, CMS, Manakamana), two hospitals, Bharatpur and CMC, were selected randomly. Later, a systematic random sampling technique with proper allocation to each hospital was used to select the study unit. The sampling interval was determined by dividing the expected number of ANC attendees per month from both hospitals and the required sample size (1780/644) with a sampling interval of three. Thus, every number based on sample interval was taken until met the required total sample size, where 75% (483) were taken from Bharatpur hospital and 25% (161) from CMC. The first Antenatal woman was selected by using the lottery method at each hospital.

#### Data collection tool

The researcher developed a semi-structured interview schedule after extensive literature review and consultation with subject experts [19–21]. The research instrument consisted of four parts: part I: Questions related to sociodemographic information, part II: Questions related to obstetric factors, part III: Questions related to awareness of COVID-19. These questions were answered on a Yes/No basis with an additional "I don’t know" option. A correct answer was assigned 1 point and an incorrect/unknown answer given 0 points. The total knowledge score ranged from 0 to 10, cutoff mean score was 8.6 higher, denoting a better knowledge of COVID-19. Part IV: Attitude Scale related to perception on acceptance of COVID-19 vaccine. These statements were answered on agreeing, neutral, and disagree basis with assigned 3 to 1 score points. It contains ten items, the total score was 30, and the cutoff mean score was 24.6, above indicates a favorable attitude.

The content validity of the tool was established by thoroughly reviewing the literature and consultation with subject experts. The tool was developed in English, translated from English to Nepali, and back-translated to English. For comprehensibility and simplicity of language, suggestions were taken by a language expert. Finally, the research instrument was pretested among 10 percent of pregnant women from different settings and did necessary modifications.

#### Ethical consideration

After getting ethical clearance from the Institutional Review Committee SMTC-IRC20220122-31, Took formal permission from the concerned authorities for data collection. Obtained written informed consent and explained the objectives of the study in clear and understandable terms. Respondents were assured that information will keep confidential by giving code numbers and informed them that they could withdraw from the study if they wished. Privacy was maintained by taking interviews separately, enclosing them with the curtain or in the corner area. Respondents were assured of no harm; every possible precaution related to COVID-19 was maintained while taking an interview. Proper sanitization to respondents and researchers was maintained. The researcher used the facemask correctly, and the respondents also advised to wear the facemask.

#### Data collection procedure

To collect data from selected ANC clinics, the researchers themselves collect the data. Data were collected by face-to-face interview method using a pretested semi-structured interview schedule among the women attending the ANC clinics of a selected hospital in Bharatpur. At the time, one participant was interviewed for 20 to 25 minutes. The researcher made every possible attempt to reduce bias in data collection. Data collectors and respondents followed the WHO COVID-19 prevention protocols such as using face masks, maintaining physical distancing, and hand sanitizer during data collection.

#### Data analysis procedure

Collected data were checked, reviewed, and organized for accuracy and completeness daily by the researcher. The responses in the completed questionnaires were coded and entered Excel 16 and exported to SPSS version 26 for analysis. It was cleaned and edited (checking for missing values and outliers). Data analysis was done using descriptive (frequency, mean, standard deviation) and inferential statistical methods (Pearson chi-square test and logistic regression, Pearson correlation). After that, the conclusion of the study was drawn and presented in tables and figures.

## Results

**Table 1**shows that out of 644 respondents, the majority, 43%, of the respondents were in the age group of 26–30 years. The mean age of respondents was 25.38 years. The minimum age was 18years, and the maximum age was 39 years. Regarding ethnicity majority, 55.3%were from Brahmin/Chettri. Consequently, most of the respondents, 81.4%, followed hinduism as their religion. At the same time, concerning education, the maximum number of respondents, 97.7%, were literate. Among them, 61.5% were from the secondary level. Concerning occupation, 82.1% were housewives, and 37.9% had 31000–60000 NRS monthly income. Most importantly, they did not have a history of COVID-19 infection in the majority, 97.4% of respondents. Similarly, 97.5% had no history of comorbidities. Furthermore, the source of information about COVID-19 and COVID-19 vaccine respondents turned out to be mass/media,i.e., 95.2%.

**Table 1:** Sociodemographic characteristics of the respondent.

			n = 644
Characteristics	Category	Frequency (%)	
Age in a group(years)	18–20	80(12.4)	
	21–25	237(36.8)	
	26–30	277(43)	
	31–35	43(6.7)	
**Mean = 25.38 SD±3.9 Min16 and Max = 39**	36–40	7(1.1)	
Ethnicity	Dalit	58(9.0)	
	Janajati	191(29.7)	
	Madhesi	11(1.7)	
	Muslim	8(1.2)	
	Brahmin/Chettri	356(55.3)	
	Others (Thakuri, Giri, Puri)	20(3.1)	
Religion	Hinduism	524(81.4)	
	Buddhism	102(15.8)	
	Islam	9(1.4)	
	Christianity	9(1.4)	
Education status	Literate	629(97.7)	
	Illiterate	15(2.3)	
If literate (n = 629)	General literate	16(2.5)	
	Basic Education	123(19.1)	
	Secondary level	396(61.5)	
	Bachelor level &above	94(14.6)	
Occupation of respondent	Service Holder	43(6.7)	
	Business	28(4.3)	
	Agriculture	34(5.3)	
	Housewife	529(82.1)	
	Wage labor	10(1.6)	
Monthly income Status (NRs)	≤ 30000	210(32.6)	
	31000–60000	244(37.9)	
	61000–90000	131(20.3)	
	>90000	59(9.2)	
History of COVID-19 infection	Yes	17 (2.6)	
	No	627(97.4)	
History of Medical Illness	Yes	16(2.5)	
	No	628(97.5)	
If Yes (n = 16)	Hypertension	9(56.25)	
	Diabetic	1(6.25)	
	Thyroid	6(37.50)	
Sources of Information (MR)	Newspaper	172(26.9%)	
	Internet and social media	576(90%)	
	FM radio	609(95.2)	
	Health worker	507(79.2)	
	Friends and Relatives	283(44.2)	

**Table 2**shows that most of the respondents, 65.1% were primigravida, and 34.9% were multigravida. Among multigravida women, 100% had a history of ANC visits during their last pregnancy; among them, 62.2% completed four and more visits. Out of 225 multigravida women, 67.6% had a history of adverse pregnancy outcomes. Concerning the week of gestation, 55.33% were from the 3^rd^ trimester. Similarly, about complications during pregnancy, 1.7% had arisen complications.

**Table 2:** Respondents obstetric related factors.

		n = 644
Characteristics	Category	Frequency (%)
Number of pregnancies (Gravida)	One	419(65.1)
	Two	186(28.9)
	Three	39(6.1)
Number of children (Para) (n = 225)	Zero(lost)	9(4)
	One	193(85.8)
	More than one	23(10.2)
History of adverse pregnancy outcome(n = 225)	No	208(92.4)
	Yes	17(7.6)
If yes, adverse pregnancy outcome (n = 17)	One time	15(88.2)
	Two time	2(11.8)
ANC visits during last pregnancy (225)	Yes	225(100)
Number of ANC visits (n = 225)	Two	8(3.6)
	Three	14(6.2)
	Four	63(28)
	More than four	140(62.2)
Complications arise during this pregnancy	No	633(98.3)
	Yes	11(1.7)
Week of gestation (Trimester)	1st Trimester	73(11.3)
	2nd Trimester	215(33.4)
	3rd Trimester	356(55.3)

**Fig 1:**
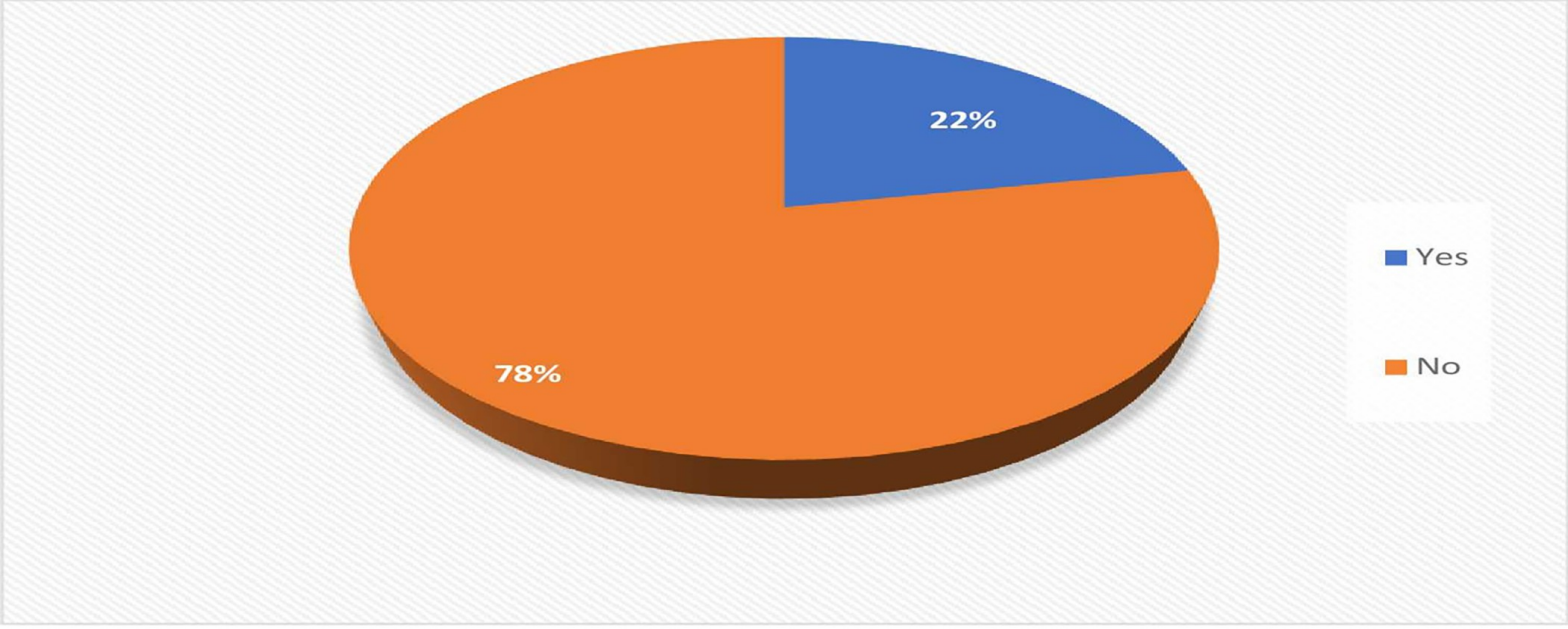
Acceptance of COVID-19 vaccine among pregnant women.

**Fig 2:**
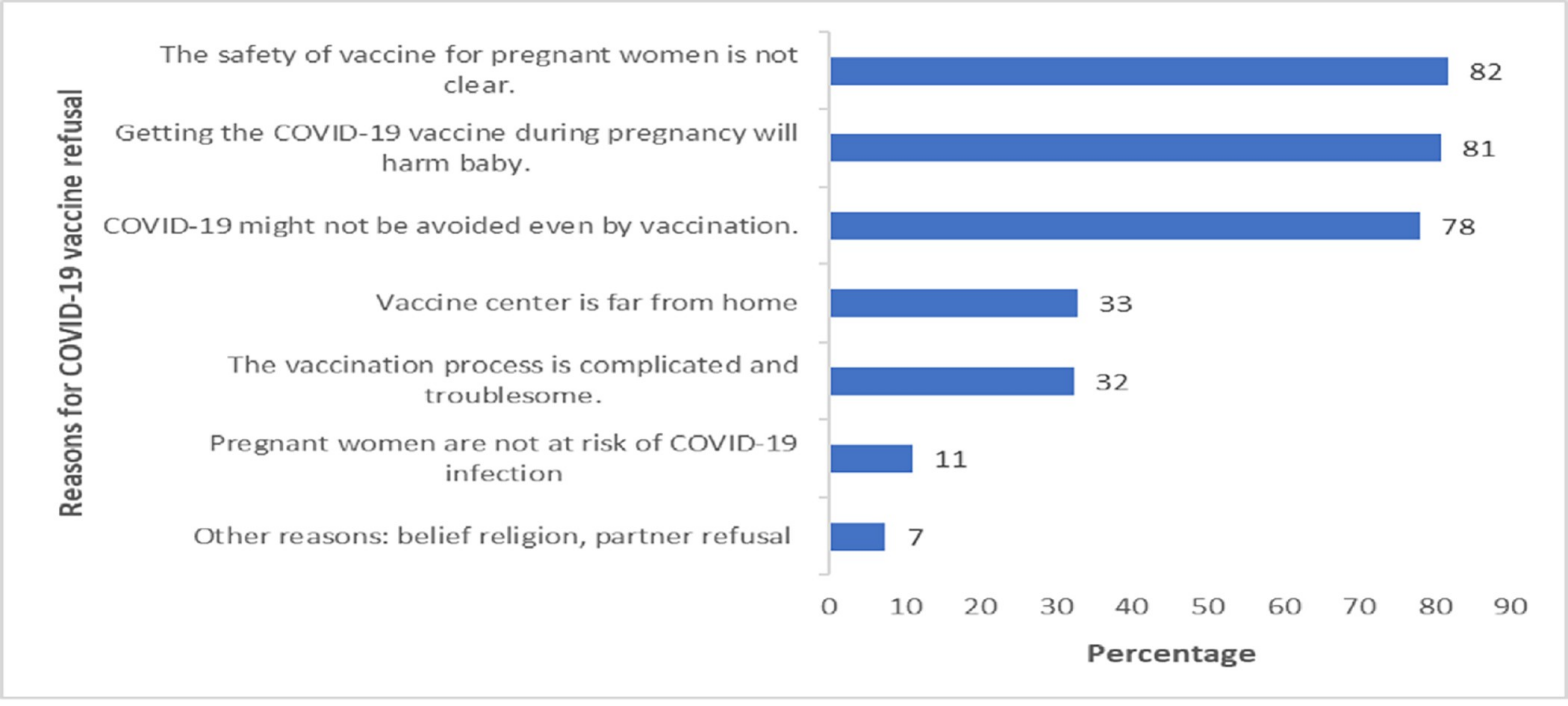
Reasons for refusal of COVID-19 vaccine by pregnant women.

**Fig 1**shows that COVID-19 vaccine acceptance was found to be 22%, and 78% of pregnant women were refused the COVID-19 vaccine. **Fig 2**shows the reasons for refusal to accept the COVID-19 vaccine, where most of the women, 82%were rejected the vaccine due to safety reasons. Other reasons for refusal were the vaccine might harm the baby 81%, the vaccine center is far 33%, vaccination process is complicated and troublesome 32%, pregnant women are not at risk 11%, and other reasons were 7%, i.e., religion belief, a family decision.

**Table 3**shows, data result delineate, that respondents socio-demographic factors such as ethnicity (AOR = 1.826; 95% CI = 1.215–2.745;p = 0.004) education level (AOR = 1.773; 95%CI = 1.025–3,068; p = 0.041), respondents monthly income (AOR = 1.755; 95% CI = 1.078–2.859; p = 0.024, respondents occupation (AOR 2.263; 95% CI = 1.183–4.329;p = 0.014), history of COVID-19 infection (AOR = 3.63; 95% CI = 1.323–9.956;p = 0.012) were found to be influential factors significant for acceptance of COVID-19 vaccine among pregnant women.

**Table 3:** Association between acceptance of COVID-19 vaccine and sociodemographic related factors.

		Acceptance of COVID- 19 Vaccine					
Variable	Category	Yes (22%)	No (78%)	UOR (95%CI)	p-value	AOR (95% CI)	p-value
Ethnicity	Brahmin/ Chettri	61(17.1)	295 (82.9)	1.892(1.298–2.758)	0.001	1.826(1.215–2.745)	**0.004**
	Other than Brahmin/Chettri	81 (28.1%)	207(71.9)	1			
Education status	Illiterate	7(46.70)	8(53.30)	3.202(1.141-.8.987)	0.027*	1	0.099
	Literate	135(21.50)	494(78.50)	1		2.629(0.917–7.536)	
Education level	<Bachelor	105(19.6)	430(80.4)	1	0.008*	1	**0.041**
	≥Bachelor	64(68.1)	30(31.9)	1.920(1.184–3.113)		1.773(1.025–3.068)	
Income	≤60,000	115 (25.3)	339(74.7)	1	0.002	1	**0.024**
	>60,000	27 (14.2)	163(85.8)	2.048(1.294–3.24)		1.755(1.078–2.859)	
Occupation	Paid	120(20.3)	471(79.7)	2.785(1.557–4.985)	0.001*	2.263(1.183–4.329)	**0.014**
	Unpaid	22(41.5)	31(58.5)	1		1	
History of COVID-19 Infection	Yes	9(52.90)	8(47.1)	4.179(1.582–11.039)	0.00**4**	3.63(1.323–9.956)	**0.012**
	No	133(21.2)	494(78.8)	1		1	
History of Medical Illness	Yes	7(43.8)	9(56.3)	2.171(0.775–6.079)	0.140	1.703(0.558–5.198)	0.35
	No	135(21.5)	493(78.5)	1		1	

Statistically significance at ≤ 0.05level, 1 = reference group, significant at 95% CI, UOR = Unadjusted odds Ratio, AOR = adjusted odds ratio.

**Table 4**showed that number of child (AOR = 5.021; 95% CI 1.989–12.677; p = 0.001), trimester (week of pregnancy) (AOR = 2.437; 95% CI 1.107–5.366; p = 0.027) and level of perception (AOR = 2.152; 95% CI 1.109–4.178; p = 0.023) were found to be statistically significant for acceptance of COVID-19 vaccine among pregnant women.

**Table 4:** Association between acceptance of COVID-19 vaccine and obstetric related factors.

		Acceptance of COVID-19 vaccine					
Variable		Yes (22%)	No (78%)	UOR (95% CI)	p-value	AOR (95% CI)	p-value
Number of children	One child	44(21.8)	158(78.2)	1			
	>One child	12(52.2)	11(47.8)	3.917(1.619–9.480)	0.002*	5.021(1.989–12.677)	**0.001**
Pregnancy complication	Yes	8(50)	8(50)	1			
	No	134(21.3)	494(78.7)	3.687(1.358–10.005)	0.010	2.344(0.236–23.257)	0.467
Trimester	1^st^ trimester	32(43.8)	41(56.2)	3.271(1.970–5.430)	0.00	2.437(1.107–5.366)	**0.027**
	2^nd^ and third trimester	110(19.3)	461(80.7)	1			
Level of perception	Favorable	81(25.8)	233(74.2)	1.533(1.053–2.232)	0.26	2.152(01.109–4.178)	**0.023**
	Unfavorable	61(18.5)	269(81.5)	1			

**Table 5**shows that the Pearson correlation test revealed a statistically significant negative correlation between perception of COVID-19 vaccine acceptance (r = −0:80; p 0.025) and a non-significant correlation between awareness of COVID-19 vaccine and acceptance of vaccine (r = -0.02 p = 0.605).

**Table 5:** Correlation between acceptance of COVID-19vaccine with awareness and perception of vaccine.

Variables	Correlation coefficient (r)	p-value
Awareness of COVID-19 vaccine–acceptance of COVID-19 vaccine	-0.02	0.605
Perception of COVID-19 vaccine–acceptance of COVID-19 vaccine	-0.80	0.025

## Discussion

Vaccination has been demonstrated repeatedly as one of the most effective interventions for preventing disease worldwide [22]. Broad distribution and acceptance of vaccines are required to achieve herd immunity and speed up the end of the pandemic [23]. Vaccination is the best way to protect against the known risks of COVID-19 in pregnancy for both women and babies [24].

The study suggested that pregnant respondents were more likely to decline vaccination compared to non-pregnant and breastfeeding respondents [25]. A similar finding in this study: only 22% pregnant women accepted, and (78%) rejected the COVID-19 vaccine. This is higher than the study finding of (13.3%) Czechia [26]. This is a low figure than the study conducted in (77.4%) China [19], (70.9%) Ethiopia [20], (62.1%) United Kingdom [27], (60.4%) Vietnam [28], and (49.1%) Japan [29] where higher acceptance of COVID-19 vaccine was found. The possible justification might be the differences in a study setting, availability of a vaccine, access to healthcare services, lack of awareness related to COVID-19, and the vaccine’s safety for both their unborn babies and themselves remained unclear. Furthermore, the time-consuming process of vaccination, belief of having a risk of infection after vaccination, and uncertainty of vaccine effectiveness were also reasons for pregnant women’s COVID-19 vaccine hesitancy [19–20].

In this study, Brahmin/Chettri ethnic groups were 1.826 times more likely to accept the COVID-19 vaccine than pregnant women from Dalit, Janajati, Madhesi, and other ethnic groups. The traditionally entrenched caste system in Nepal has been a barrier to the ability of some castes and ethnic populations to access health care services. Caste differentials concerning health status in which Dalit and Janajati women have lower utilization of essential health care services [30]. A possible explanation among higher caste Brahmin/Chettri might be indicating a higher knowledge, income, education, and the higher societal value given to this caste group may have the greater acceptance.

This study found more acceptance of the COVID-19 vaccine among the educated, higher level of education had 1.773 times more likely to accept the COVID-19 vaccine than pregnant women who had lower education levels. The finding was consistent with a recent study that showed that years of education increase, and so does the acceptance of a COVID-19 vaccine [21,31]. The possible explanation might be education makes mothers be more concerned for their health and have more autonomy to make decisions about their health. Educated mothers might be more aware of COVID-19 related prevention and have the ability to read books and news and follow social media related to the COVID-19 virus impact [28,29]. This is contradictory to the findings of the study conducted in China [19] suggested that pregnant women in western China with higher education levels had higher vaccine hesitation and low education level had higher COVID-19 vaccination intentions. This might be due to education eventually enhancing their knowledge of the COVID-19 vaccine, and they might get more negative information about the COVID-19 vaccine [31]. Therefore, this suggested that evidence-based vaccine research is required to reduce public concern about vaccine safety.

In this study, those in paid occupations had 2.263 times higher acceptance of the COVID-19 vaccine. This finding is supported by a study conducted in Saudi [21], where the pregnant woman involved in a paid occupation was associated with higher acceptance of the COVID-19 vaccine compared to unpaid or being a housewife. Additionally, various studies showed higher monthly income greater the acceptance of vaccines [21,28,29,31].

This study finding showed that the higher the monthly income, 1.755 times higher the acceptance of the covid vaccine. Additionally, many studies have shown higher monthly income is more significant in acceptance of vaccines [20,25,29,31,32]. This might be due to the low-income communities being generally concentrated in remote areas with low income and education, which hinders access to essential health care services.

According to WHO the immunity people get from being vaccinated after having a natural infection is consistently very strong. Getting vaccinated even before the history of COVID-19 means more likely to be protected for longer [33]. In this study, the women who had a history of COVID-19 infection found 3.63 times higher acceptance of the COVID19 vaccine. This might be due to previous experiences, knowledge of prevention, fear of complications, and counseling from health workers.

Symptomatic pregnant women with comorbidities and COVID-19 have an increased risk for severe maternal outcomes compared to non-pregnant women, including risk for intensive-care unit (ICU) admission, invasive ventilation, and even death [34,35]. It was expected that women at a higher risk for severe COVID-19 due to comorbidities might have a higher level of vaccine acceptance. But surprisingly, in this study who had a history of comorbidities had less likelihood of accepting the COVID-19 vaccine. This finding is supported by a study [36] which reveals having comorbidities that would increase one’s risk for COVID-19 did not impact women’s willingness to receive a vaccine. A possible explanation may be that pregnant women with comorbidities think that the vaccine has the possibility of harming their and babies’ health and increasing the risk for adverse pregnancy outcomes [28].

A pregnant mother for the second time and who had child 5.021 times were found to have higher acceptance of the COVID-19 vaccine. This finding is supported by [32] and contrast with the study conducted in China [19]. This study reveals the low level of COVID-19 vaccine acceptance among pregnant women and mothers of young children. The possible explanation might be that women who were pregnant for a second time or had one child are usually more likely to have less fear of childbirth, experience, and knowledge from previous pregnancies and births. In contrast, primipara women were more conscious of pregnancy and feared complications and side effects of the vaccine [37].

In this study, women who had in the 1^st^ trimester were found 2.437times more acceptance of covid vaccine than the 2^nd^ and 3^rd^ trimester. These findings were consistent with Goncu Ayhan et al. [38], which showed pregnant women in the second and third trimesters expressed lower vaccine acceptance than those in the first trimester. But these findings were in contrast with the results of the study conducted in China [19]. Studies have found that fear, anxiety, and depression are prevalent symptoms in the first trimester of pregnancy, and the first trimester of pregnancy during the COVID-19 pandemic may be more distressed than in prior pregnancies. This level of anxiety may lead to greater acceptance of COVID-19 vaccination in the first trimester of pregnancy and high-risk pregnant women [38,39]. The COVID-19 vaccines should be effective at any stage of pregnancy. Pregnant women are more likely to become seriously unwell when compared to non-pregnant women and have a higher risk of their baby being born prematurely if they develop COVID-19 in their third trimester (after 28 weeks of gestation). Therefore, it is reasonable to aim to have the vaccine before the 2^nd^ third trimester [40].

In this study, the good knowledge level of pregnant respondents, such as the source of infection, route of transmission, risk population, major clinical features, focused preventive measures, etc. Surprisingly, our study found a negative correlation between total knowledge score in COVID-19 and acceptance of COVID-19 vaccines. This finding was contrary to the study findings [19,20], where a positive correlation between knowledge and acceptance were found. That might be due to pregnant women with a high level of knowledge related to COVID-19 having more access to various information, which may increase the likelihood of receiving incomplete, inaccurate information, leading to their potential misunderstanding of the efficiency and safety of a vaccine [41].

Similarly, the finding of this study showed that a favorable perception toward the vaccine of COVID-19 had 2.152 times higher acceptance of the COVID-19 vaccine than an unfavorable perception. This finding is supported by the result of this study conducted in Ethiopia [20] revealed that more than half, 66.7% of pregnant women had a good attitude towards COVID-19. This could be because pregnant women might strictly follow the health care workers’ counseling/advice during their antenatal care visit regarding COVID-19 preventive measures [38].

In this study, the reason for unwillingness to COVID-19 vaccine was (81.8%) the vaccine’s safety for pregnant women. Similarly, the primary reason for vaccine rejection was the absence of evidence on COVID-19 vaccine safety, consistent with a previous study published in China [19,39,42]. Several implications would be proposed according to our findings. This can be explained by the natural fear of a mother toward her pregnancy. As more data become available, there will be more opportunity to build trust in the scientific approval of these vaccines [19–29]. The second-most common reason was a belief that the vaccine might harm their babies were less likely to accept vaccination (80.8%) which was further supported by various studies [38].

The study was confined to the selected hospital of Bharatpur Chitwan only, so findings cannot be generalizable in all places. COVID-19 pandemic-related issues itself a challenge to conduct the hospital-based study. Confounding bias may occur due to unmeasured variables.

## Conclusion

In this study, only 22% of pregnant women accept COVID-19 vaccination during pregnancy. Ethnicity, education level, income, occupation, history of COVID-19 infection, number of pregnancies, week of gestation, and status of perception were influential factors significant for acceptance of the COVID-19 vaccine among pregnant women. Vaccinated people are protected from getting the disease and breaking any chains of transmission. But Vaccine hesitancy is one of the major threats to the COVID-19 vaccine rollout and successful mitigation of the pandemic. Vaccine coverage is required for herd immunity So, everyone needs vaccine acceptance to get herd immunity and lessen the COVID-19 infection. COVID-19 vaccination program helps reduce infections and deaths and likely reduce overall health care costs. Therefore, properly disseminating information and removing misperceptions about the COVID-19 vaccine is necessary to raise the acceptance.

## Supporting information

S1 Table Respondents’ awareness of COVID-19 related factors. (DOCX)

S2 Table Respondents’ perception of COVID-19 vaccine. (DOCX)

S1 File Data available: Data are available in OSF: https://doi.org/10.17605/OSF.IO/2B376. (DOCX)
