# Supplementary material for: Pregnant women’s awareness, perception, and acceptability of COVID-19 vaccine attending antenatal clinics in Bharatpur, Nepal
Source: PLoS One. 2023 Mar 15;18(3):e0278694. doi: 10.1371/journal.pone.0278694 (PMC10016669; doi:10.1371/journal.pone.0278694)
Supplement: S1 Table — (DOCX) [file pone.0278694.s001.docx]

**Supporting Information**

| **S1 TABLE** | | |
| --- | --- | --- |
| **Respondents' Awareness of COVID-19 related Factors** | | |
|  |  | **n=644** |
| **Awareness Items** | **Correct Response** | **Frequency (%)** |
| COVID-19 is a viral infection | Yes | 637(98.9) |
| Major Transmission | Handshaking | 511(79.3) |
|  | Sitting nearby with an infected person | 596(92.5) |
|  | Body fluid of | 428(66.5) |
|  | Cough of an infected person | 636(98.8) |
| Main three features of COVID-19 | Fever, cough, and malaise | 639(99.2) |
| Focused Preventive measures | Maintaining a distance of at least 1 meter between everyone | 335(52) |
|  | Wearing a well-fitting face mask. | 637(98.9) |
|  | Washing hands with soap and water, and using disinfectants. | 630(97.8) |
|  | Taking a complete dose of vaccine | 498(77.3) |
| Level of knowledge | Good (8.7-10) | 366(56.8) |
|  | Poor (1-8.6) | 278(43.2) |

**S1Table-** shows that out of 644 respondents, the majority, 56.8% respondents had good knowledge regarding COVID-19; among them, 98.9% of the respondents knew the meaning of COVID-19; regarding direct transmission, 79.3% gave the correct response. The majority of the respondents, 99.2%, showed a correct answer on the most common signs and symptoms. Concerning preventive measures, 98.9% responded correctly to wearing a well-fitting face mask.
