# Supplementary material for: Pregnant women’s awareness, perception, and acceptability of COVID-19 vaccine attending antenatal clinics in Bharatpur, Nepal
Source: PLoS One. 2023 Mar 15;18(3):e0278694. doi: 10.1371/journal.pone.0278694 (PMC10016669; doi:10.1371/journal.pone.0278694)
Supplement: S2 Table — (DOCX) [file pone.0278694.s002.docx]

| **S2 TABLE** | | | |
| --- | --- | --- | --- |
| **Respondents’ Perception of COVID-19 Vaccine** | | | **n=644** |
| **Statements** | **Agree** | **Neutral** | **Disagree** |
| Vaccines are important to protect others in the community | 504(78.3) | 52(8.1) | 88(13.7) |
| Vaccines protect me from disease | 568(88.2) | 28(4.3) | 48(7.5) |
| I trust the information I receive about vaccines | 577(89.6) | 28(4.3) | 39(6.1) |
| I am worried that vaccines cause birth defects and other long-term negative effects on the neonate | 595(92.4) | 15(2.3) | 34(5.3) |
| I don’t believe vaccines are safe during pregnancy | 51(7.9) | 21(3.3) | 572(88.8) |
| There are too many side effects associated with vaccines | 542(84.2) | 25(3.9) | 77(12) |
| The ingredients in vaccines are harmful | 272(42.2) | 161(25) | 211(32.8) |
| Vaccines are not necessary to prevent the spread of disease | 372(57.8) | 93(14.4) | 179(27.8) |
| I trust that Pregnant women who receive the COVID-19 vaccine can prevent their babies from COVID-19 infection | 211(32.8) | 128(19.9) | 305(47.4) |
| The vaccine will help to end COVID-19 pandemic | 218(33.9) | 152(23.6) | 274(42.5) |

**S2Table-**shows that 78.3% of respondents agree that vaccines are essential to protect others in the community, paradoxically 88.2% of respondents agree that vaccines protect me from disease. Same time, 89.6% of respondents agree that they trust the information received about vaccines. In addition, 92.4% of respondents agreed that they are worried that vaccines cause birth defects and other long-term adverse effects on the neonate. However, 88.8% of respondents disagree that vaccines are safe in pregnancy. On the contrary, 84.2 of respondents agreed there are too many side effects associated with vaccines. Likewise, 42.3% of respondents agree that the ingredients in vaccines are harmful. Likewise, 57.8% agree vaccines are not necessary to prevent the spread of disease. Similarly, 47.4% disagree with pregnant women who receive COVID-19; the vaccine can prevent their babies from COVID-19 infection. Finally, 42.5% of respondents disagreed that vaccines help to end COVID-19 pandemic.
