# Supplementary material for: Pregnant women’s awareness, perception, and acceptability of COVID-19 vaccine attending antenatal clinics in Bharatpur, Nepal
Source: PLoS One. 2023 Mar 15;18(3):e0278694. doi: 10.1371/journal.pone.0278694 (PMC10016669; doi:10.1371/journal.pone.0278694)
Supplement: S1 File — (DOCX) [file pone.0278694.s003.docx]

**S1 Data Available:** Data are available in OSF: **https://doi.org/10.17605/OSF.IO/2B376**
